# Carcinogenicity of intermediate frequency magnetic field in Tg.rasH2 mice

**DOI:** 10.1002/bem.22177

**Published:** 2019-03-15

**Authors:** Izumi Nishimura, Yuko Doi, Norio Imai, Mayumi Kawabe, Yukinori Mera, Takeo Shiina

**Affiliations:** ^1^ Central Research Institute of Electric Power Industry Abiko Japan; ^2^ DIMS Institute of Medical Science Ichinomiya Japan; ^3^ Central Research Institute of Electric Power Industry Yokosuka Japan

**Keywords:** short‐term bioassay, health risk assessment, induction heating, 20‐kHz, wireless power transfer system

## Abstract

Although the likelihood of exposure to leaking intermediate frequency magnetic fields (MFs) from electronic devices, such as induction‐heating and wireless power transfer systems, has increased, biological data assessing the health risks associated with human exposure remain insufficient. We examined the carcinogenicity of a 20 kHz MF, a typical frequency produced by induction‐heating cookers, using a transgenic rasH2 mouse model. Twenty‐five male and female CByB6F1‐Tg(HRAS)2Jic mice were exposed to a 0.20 mT, 20 kHz MF (22 h/day) or sham‐exposed for 26 weeks. As a positive control, 10 male and female rasH2 mice from the same batch were administered a single intraperitoneal injection of 75 mg/kg *N*‐methyl‐*N*‐nitrosourea. A blinded histopathological evaluation was performed, and the same experiments were conducted twice, independently, to confirm the reproducibility of the results. Histopathological examination revealed that spontaneous neoplastic lesions, such as splenic hemangiosarcomas and gastric squamous cell papillomas, were less (1–3 per group) in the MF‐ and sham‐exposed groups. The frequency of the neoplastic lesions was not significantly different between the groups. Eight to ten mice in each positive‐control group exhibited malignant lymphoma. The outcomes were consistent between duplicated experiments, which indicates lack of carcinogenicity of 20 kHz MF in the rasH2 mouse model. Bioelectromagnetics. © 2019 The Authors. *Bioelectromagnetics* Published by Wiley Periodicals, Inc.

## INTRODUCTION

The chances of people encountering intermediate frequency (IF) magnetic fields (MFs) leaking from domestic instruments and industrial machines utilizing IF‐MFs have increased in recent times. IF‐MFs, with a frequency range from 300 Hz to 10 MHz, are used in various electronic devices, such as induction‐heating cookers (20–90 kHz) and wireless power transfer systems (20 or 85 kHz) in electric vehicles [Yamazaki et al., 2004; Fujita et al., 2010; Tell et al., 2014]. In domestic situations, induction‐heating cookers have been reported to be among the relatively intense sources of exposure [Leitgeb et al., 2008; Christ et al., 2012; Aerts et al., 2017]. However, the IF‐MF intensity decreased rapidly with distance from the hob of cookers [Viellard et al., 2007; Federal Office of Public Health (FOPH), 2016]. Emission measurements and human model computations [Kos et al., 2011] indicated that IF‐MFs from domestic induction‐heating cookers sufficiently conform to the International Commission of Non‐Ionizing Radiation Protection (ICNIRP) guidelines [2010]. The maximum exposure of fetuses in utero was found to be less than 2% of the guidelines’ value. In the latest assessment, the IF‐MF intensities to which children are exposed in daily life were less than 1% of the value of the guidelines [Gallastegi et al., 2017].

The World Health Organization (WHO) has reviewed numerous papers regarding the biological effects of exposure and has conducted health risk assessments of low‐frequency MFs. Environmental Health Criteria 238 (EHC238) [WHO, 2007] has stated that more research is required to conduct health risk assessments. Following the EHC238, the Scientific Committee on Emerging and Newly Identified Health Risks (SCENIHR) reported an updated risk assessment [SCENIHR, 2015]. Some new evidence regarding the health hazards of IF‐MF included reproductive and developmental toxicity, genotoxicity, and other cellular responses. Although two carcinogenicity studies [Svedenstål and Holmberg, 1993; Lee et al., 2007] were published prior to the SCENIHR report, new carcinogenicity studies have yet to be reviewed.

The present study investigated the potential carcinogenicity of exposure to 20 kHz MFs. We used the transgenic rasH2 mouse model, as it allows detection of the effects of both genotoxic and nongenotoxic carcinogens [Yamamoto et al., 1998; Morton et al., 2002]. The model has also been internationally approved as an alternative to the two‐year rat carcinogenicity bioassay [ICH, 1997] and is presently one of the most frequently used mouse models for carcinogenicity studies [Bourcier et al., 2015; Hickman, 2015], with applications extending beyond pharmaceuticals [Palazzi and Kergozien‐Framery, 2009; Takanashi et al., 2012; Doi et al., 2017]. The 20 kHz is a typical heating frequency for induction‐heating cookers and is used in some wireless power transfer systems for electric vehicles [Tell et al., 2014]. The intensity of the MF (200 μT(root‐mean‐square: rms)) was approximately 7.4 times higher than the ICNIRP guidelines. It was the highest intensity possible in our animal exposure facility and was in accordance with the methodology of our series of previous rodent toxicity studies [Nishimura et al., 2011, 2012, 2016]. The waveform of the MF was sinusoidal to allow clearly defined and replicable exposure conditions. The exposure was continuous to maximize the total exposure time. The experiment was duplicated in different batches of animals, as the present study was comprised of groups exposed to only one dose of MF and sham radiation (control), unlike the standard four doses of toxicological testing regimes. The baselines of the control and positive control groups had to be defined and reproducibility of the experimental outcomes could be confirmed. After MF exposure, the histopathology was examined in a good laboratory practice (GLP)‐licensed laboratory under blinded exposure conditions for each animal.

## MATERIALS AND METHODS

### MF Exposure and Dosimetry

A group of mice was exposed to a vertically polarized 20 kHz MF at 200 μT(rms) with a sinusoidal waveform for 26 weeks, whereas another group of sham‐exposed animals received an extremely low intensity (less than 0.001 μT(rms)) of background IF‐MF. The background 50 Hz MF was 0.03 μT(rms) and the geomagnetic field was approximately 48.3 μT. Details of the exposed MF and animal exposure facility were explicitly described in previously published papers with a photograph [Nishimura et al., 2011, 2012]. In brief, the pair of sham‐exposure and MF‐exposure facilities was located 37.4 m apart to eliminate any stray IF‐MF from energized coils placed in the MF‐exposure facility. Merritt‐type coils having four horizontally‐layered squares [Merritt et al., 1983] produced very uniform MF at the center cubic space (100 × 100 × 100 cm) of the coils, where 25 male and 25 female mice were housed on three wooden racks [Nishimura et al., 2011]. Spot measurements at the animal exposure space were performed using a commercial instrument (ELT‐400; Narda Safety Test Solutions, Pfullingen, Germany) with a 100 cm^2^ probe and showed that the spatial variability of the MF flux density was less than 3%. The harmonic components of the MF, variability of the electric current in the coils, and emission of ultrasound noise, vibration, and heat from the coils were all negligible. Effects of the animal housing equipment, for example, polyolefin cages and wood chips, on the intensity and uniformity of the MF exposure were undetectable. The sham‐exposed facility was equipped with the same coils and wooden racks, and had a similar appearance; however, the coils were not energized. During the exposure term, the 20 kHz electric current in the exposure coils was continuously monitored and recorded every 10 min. The time variability of the current was less than 0.2%.

For dosimetry, the mouse was assumed to be a homogeneous spheroid to calculate the induced electric field as whole body exposure. The calculation was conducted based on Reilly's equation [Reilly, 1998] indicated below, where 2*a* and 2*b* were defined as the major and minor axes of the elliptical cross‐section of the spheroid, respectively.

$$E_{\max}=\frac{2\pi fBa^{2}b}{a^{2}+b^{2}}$$

The mouse body including the head was approximated to have a major axis of 8.0 cm and a minor axis of 3.6 cm. The maximum induced electric field (*E*_max_) occurred on the periphery where the minor axis intersected the elliptical cross‐section. The derived electric field was 0.38 V/m for the exposure to 0.20 mT, 20 kHz MF perpendicular to the cross‐section of the mouse body when the mouse was in lying‐down posture.

Recently, Kumari et al. [2017] calculated the induced electric field in the organs of a C57BL/6J male mouse that was exposed to a 7.5 kHz, 120 μT MF at the maximum, based on a model developed by the Swiss Federal Institute of Technology [IT'IS Foundation, 2017]. Based on the data presented in their paper, we made a rough calculation of the induced electric fields in the organs and tissues of mice used in the present study. The induced electric fields in Kumari's study were scaled up to the MF intensity of our study (0.20 mT). When tissue conductivity is not a function of MF frequency, the induced electric fields are proportional to the frequency of the incident MFs [Gandhi and Chen, 1992]. We therefore scaled up the electric fields *E* to the frequency *f* (20 kHz) of interest as shown below.

$$E=\frac{f}{f^{\prime }}E^{\prime }$$

*E*′ and *f*′ were the induced electric fields and the frequency of Kumari's study, respectively. The calculation resulted that a relatively small electric field could be induced in the urethra, thymus, and tendons (0.07, 0.07, and 0.09 V/m, respectively), whereas relatively large values were found in the skin, bones, and fat (1.0, 0.76, and 0.71 V/m, respectively). Based on the parametric model proposed by Gabriel et al. [1996], we confirmed that differences in the tissue conductivities between 7.5 kHz and 20 kHz were small.

### Animal Husbandry and Experimental Design

The Institutional Animal Experiment Committee of the Environmental Science Research Laboratory, Central Research Institute of Electric Power Industry, Japan, as well as DIMS Institute of Medical Science, Japan, approved all the animal experimental procedures used in the present study in accordance with the Committee's guidelines under the Japanese Law Concerning the Protection and Management of Animals. Referring to the ICH‐S1 Harmonised Tripartite Guideline for testing carcinogenicity [ICH, 1997], the study was performed using the short‐term in vivo rodent test. The exposure of animals to IF‐MFs was conducted at the Central Research Institute of Electric Power Industry while the positive control treatment was conducted at the DIMS Institute of Medical Science, using the same batch of animals. Complete autopsy and pathological examinations were conducted by personnel from the DIMS Institute of Medical Science, under blind conditions. Each animal was randomly numbered and coded by the former institution and then decoded at the latter institution after completion of the histopathological examination.

Two independent and identical experiments (Experiments I and II) were conducted. For each experiment, six‐week‐old male and female (63 each) CByB6F1‐Tg(HRAS)2Jic mice (rasH2 mice) were purchased from CLEA Japan (Tokyo, Japan). After 1 week of quarantine, the mice were randomly assigned to the MF‐exposed, sham‐exposed (control), or *N*‐Methyl‐*N*‐nitrosourea (MNU)‐treated (positive control) groups with 25, 25, and 10 mice per sex, respectively. The MF exposure was initiated when the animals were seven‐weeks old, and at the same time the positive control groups received a single intraperitoneal administration of 75 mg/kg body weight (bw) MNU (Sigma–Aldrich Japan, Tokyo, Japan) dissolved in citrate‐buffered saline (pH 4.5), as 10 ml/kg bw.

The animal exposure facilities had a barrier system with HEPA‐filtered air; the room temperature was 23 ± 2 °C, humidity was 50 ± 20%, and light was provided in a 12/12 h cycle (on from 07:00 to 19:00 h). Autoclaved polyolefin mouse cages (14 cm W × 29 cm D × 15 cm H; Charles River Laboratories Japan, Kanagawa, Japan), with wood chips, polycarbonate cage‐top, and a water bottle with glass mouthpiece were used for individual housing. Irradiated pellets (CLEA Japan) and water were provided ad libitum. The MF exposure lasted 22 h/day (11:00–09:00 h), and animal care and data collection were performed every day for 2 h. The positions of the animal cages were changed every week to equalize subtle differences in environmental conditions and MF intensity. The same experimental regime was applied to the sham‐exposed animals. The MNU‐treated animals were also housed in a barrier system under similar air, lighting, and other conditions in a separate animal facility at the DIMS Institute of Medical Science.

## MEASUREMENTS AND EXAMINATIONS

### Morbidity, Clinical Observation, and Body Weight

Clinical observation of each animal was performed every day and the findings were recorded. The body weight was measured every week and recorded. Based on the prescribed humane endpoints, the morbid animals were euthanized followed by necropsy and further examinations. Animals found dead were also necropsied, grossly examined, and their organs and tissues were excised and fixed for histopathological examination. At the termination of exposure, all the surviving animals were anesthetized by isoflurane inhalation and blood was drawn from the abdominal aorta to sacrifice the animals humanely by exsanguination.

### Gross Pathology and Organ Weight

After necropsy, organs and tissues were grossly examined and the findings were recorded. The multiplicity of some countable abnormal findings, such as nodules, was graded. The following organs and tissues were excised for further examination: heart, aorta, lymph nodes (mandibular and mesenteric), spleen, bone marrow, thymus, pituitary, thyroid, parathyroid, adrenals, nasal cavity, trachea, lung/bronchial, tongue, salivary gland, esophagus, stomach, duodenum, jejunum, ileum, cecum, colon, rectum, pancreas, liver, gall bladder, kidneys, urinary bladder, testes, prostate, epididymis, seminal vesicle, mammary gland, ovaries, uterus, vagina, femur, sternum, musculature, skin/subcutis, eyes, Harderian gland, brain, spinal cord, sciatic nerve, and lacrimal gland. These samples were fixed in 10% neutral‐buffered formalin solution for histopathological examination. The solution was gently infused into some organs, such as the trachea, lung, stomach, intestines, and urinary bladder. If a tissue that was not intended to be excised was found to have pathological changes, it was also fixed for histopathological investigation.

The brain, heart, kidneys, liver, testes, ovaries, spleen, thymus, and lung were weighed before fixing, and the organ weight relative to the body weight was calculated. The organs from animals that were found dead, morbid, or in the MNU‐treated group were not weighed.

### Histopathology

Histopathological examination was conducted on all the samples of fixed organs and tissues procured from all animals. The samples were embedded in paraffin, sectioned, and stained with hematoxylin and eosin for microscopic examination. The observed histopathological changes were recorded and the malignancy/benignancy of the lesions was judged based on their severity, metastasis, and invasion into other organs and tissues. Some non‐neoplastic lesions, such as hematopoiesis and myopathy, were graded by severity.

### Statistics

Statistical analyses were performed between MF‐exposed and sham‐exposed groups or between positive control and sham‐exposed groups, on animals of both sexes, using commercial software (StatLight 2000; Yukms, Kanagawa, Japan); the significance threshold values were *P* < 0.05 or *P* < 0.01. The survival rate was assessed using the log‐rank test. The body weight, hematological parameters, and organ weight relative to the body weight were presented as group means ± SD, and were first assessed for data homogeneity using an *F*‐test. When the group variances were equal, Student's *t*‐test (one‐tailed test) was used; when the variances were not equal, Welch's *t*‐test (one‐tailed test) was used. Fisher's exact test (one‐tailed test) was applied to evaluate group differences in gross pathological and histopathological findings, whereas countable findings and graded findings were analyzed using Wilcoxon's test (two‐tailed test).

## RESULTS

### Survival and Body Weight

The survival data are summarized in Table 1. In the sham‐exposed groups, which included male and female mice, the survival percentage was 96% (24/25 mice) at the termination of the 26‐week experiments; the MF‐exposed groups ranged from 100% to 88% (22/25 mice; females in Experiment II). In the MNU‐treated group, the first dead/morbid animal was found at 10 (males in Experiments I and II) to 12 weeks (females in Experiment I) into the experiments and the final survival rates were 20% (males in Experiment I) to 0%. No statistically significant difference was found between the MF‐exposed and concurrent sham‐exposed groups at the termination of experiments.

**Table 1:** Survival Data of rasH2 Mice Exposed to a 20 kHz Magnetic Field

					Experiment I			Experiment II		
Sex	Group	Dose	No. of animals	Duration of experiment (weeks)	First dead mouse (weeks)	Terminal no. of surviving		First dead mouse (weeks)	Terminal no. of surviving	
Male	Sham	0 mT	25	26	23	24	(96%)	21	24	(96%)
	MF Exp	0.20 mT	25	26	N/A	25	(100%)	16	23	(92%)
	MNU	75 mg/kg	10	26	10	2	(20%)	10	0	(0%)
Female	Sham	0 mT	25	26	11	24	(96%)	26	24	(96%)
	MF Exp	0.20 mT	25	26	N/A	25	(100%)	15	22	(88%)
	MNU	75 mg/kg	10	26	12	0	(0%)	11	0	(0%)

Sham, sham‐exposed; MF Exp, magnetic field‐exposed; MNU, *N*‐methyl‐*N*‐nitrosourea‐treated.

The sham‐ and MF‐exposed mice grew normally. The group means for the body weight gradually increased from the first week toward the end of the 26‐week experiment. Over the period of the experiment, the body weight of male mice in the MF‐exposed group was slightly higher compared to that in the sham‐exposed group in Experiment I. The differences in the group mean, by week, were small (1.65 g being the largest difference) and occasionally statistically significant. The changes were, however, opposite between Experiments I and II. The curves for the body weight of the female mice in the MF‐ and sham‐exposed groups were almost identical in Experiments I and II. The MNU‐treated groups of male and female mice showed a decrease (mostly statistically significant) in body weight compared to the sham‐exposed groups throughout the period of the experiment. The group means often fluctuated because the morbid mice were lean.

### Organ Weights

The absolute organ weight and organ weight relative to body weight of the surviving animals are provided in supplementary Tables S1 and S2. Compared to that in the sham‐exposed group, increases in absolute weight in male MF‐exposed mice were found in the liver, kidneys, heart, and lung in Experiment I, as well as the heart of female mice in Experiment II. Similar changes were observed in the relative weight except for the lung. A statistically significant increase in the relative weight of the liver in the MF‐exposed male mice was found in both Experiments I and II; the differences in weight (in g/100 g bw) for the sham‐exposed versus MF‐exposed groups were 4.01 versus 4.18 and 3.95 versus 4.11 in Experiments I and II, respectively.

### Gross Pathological Findings

The gross pathological findings in male and female mice are shown in supplementary Tables S3 and S4, respectively. The graded multiplicity of some findings is shown collectively, and findings with a frequency of less than two per group have been excluded from the tables. As is evident from the tables, no remarkable findings were observed in the sham‐ and MF‐exposed groups. Discolored areas/spots in the spleen and lung were occasionally noted in 0–5 mice per group; however, there was no significant increase in the MF‐exposed groups. In the MNU‐treated groups, the dead mice contained fluid in their thoracic cavity. Nodules in the spleen, forestomach, small and large intestine, and liver, and enlarged lymph nodes, spleen, and thymus were also associated. Some of these events were shown to be significantly increased when compared to those in the sham‐exposed group.

### Histopathological Findings

#### Neoplastic Lesions

Neoplastic lesions present in the male and female mice are shown in Tables 2 and 3, respectively. The frequency of neoplastic findings was low across the MF‐ and sham‐exposed groups. The most frequent findings (1–3 mice per group) were splenic hemangiosarcomas, gastric squamous cell papillomas, and Harderian gland adenomas, followed by pulmonary bronchiolo‐alveolar adenomas, cutaneous squamous cell papillomas, and ovary hemangiomas. Malignant lymphoma was also found in one male mouse in the sham‐exposed group and in two female mice in the MF‐exposed groups, in both cases in Experiment I. None of these findings showed a statistically significant difference between the MF‐exposed and concurrent sham‐exposed groups. A hitherto unreported lesion, namely carcinoma of the clitoral gland, was found in an MF‐exposed female mouse in Experiment I; however, it was only one incidence and did not occur in Experiment II. In the MNU‐treated groups, most of the animals (ranging from 8 to all 10 mice per group) exhibited malignant lymphomas. Gastric squamous cell papillomas and carcinomas were also major findings and were significantly increased compared to the sham‐exposed mice; these were followed by incidences of pulmonary bronchiolo‐alveolar adenomas, small intestinal adenomas and adenocarcinomas, cutaneous squamous cell papillomas, and others.

**Table 2:** Neoplastic Lesions of Male rasH2 Mice Exposed to a 20 kHz Magnetic Field

		Experiment	Experiment I			Experiment II		
		Group	Sham	MF Exp	MNU	Sham	MF Exp	MNU
Organ	Findings	Dose	0 mT	0.20 mT	75 mg/kg	0 mT	0.20 mT	75 mg/kg
No. of animals/group			25	25	10	25	25	10
Spleen								
	Hemangioma		0	0	0	1	0	0
	Hemangiosarcoma		3	0	1	1	0	1
Bone marrow								
	Hemangioma		0	1	0	0	0	0
Thymus								
	Hemangioma		0	0	1	0	0	0
Nasal cavity								
	Carcinoma, squamous cell		0	0	1	0	0	0
Lung/bronchial								
	Adenoma, bronchiolo‐alveolar		2	0	1	0	0	1
Stomach								
	Papilloma, squamous cell		1	1	9**	0	1	7**
	Carcinoma, squamous cell		0	0	1	0	0	4**
Jejunum								
	Adenoma		0	0	2	0	0	0
	Adenocarcinoma		0	0	2	0	0	1
Ileum								
	Adenoma		0	0	1	0	0	1
Cecum								
	Adenocarcinoma		0	0	0	0	0	1
Rectum								
	Adenoma		0	0	1	0	0	0
Liver								
	Hemangioma		1	0	0	0	0	0
Urinary bladder								
	Hemangioma		0	0	0	1	0	0
Testis								
	Hemangioma		0	0	0	1	0	0
Epididymis								
	Hemangioma		1	0	0	0	0	0
Musculature								
	Hemangioma		0	0	0	0	1	0
Skin/subcutis								
	Hemangioma		0	0	1	0	0	0
	Papilloma, squamous cell		0	0	1	0	0	0
	Carcinoma, squamous cell		0	0	0	0	0	1
Brain								
	Hemangioma		1	0	0	0	0	0
All sites			[1]^a^		[8]^a^			[8]^a^
	Malignant lymphoma		1		8			8

Sham, sham‐exposed; MF Exp, magnetic field‐exposed; MNU, *N*‐methyl‐*N*‐nitrosourea‐treated.

^a^ Numbers in square brackets are for animals examined microscopically.

^*^Significant difference compared to the sham‐exposed group (*P* < 0.01).

**Table 3:** Neoplastic Lesions of Female rasH2 Mice Exposed to a 20 kHz Magnetic Field

		Experiment	Experiment I			Experiment II		
		Group	Sham	MF Exp	MNU	Sham	MF Exp	MNU
Organ	Findings	Dose	0 mT	0.20 mT	75 mg/kg	0 mT	0.20 mT	75 mg/kg
No. of animals/group			25	25	10	25	25	10
Mesenteric lymph node								
	Hemangioma		1	0	0	0	0	0
Spleen								
	Hemangioma		0	0	0	0	0	1
	Hemangiosarcoma		2	0	1	3	1	0
Bone marrow								
	Hemangioma		0	1	1	1	0	0
Thymus								
	Hemangioma		0	0	2	0	0	0
	Hemangiosarcoma		1	0	0	0	1	0
Lung/bronchial								
	Adenoma, bronchiolo‐alveolar		0	0	0	0	0	1
	Adenocarcinoma, bronchiolo‐alveolar		0	0	0	0	1	0
Tongue								
	Papilloma, squamous cell		0	0	1	0	0	0
Salivary gland								
	Carcinoma, squamous cell		0	0	0	0	0	1
Esophagus								
	Papilloma, squamous cell		0	0	0	0	0	1
Stomach								
	Papilloma, squamous cell		0	3	8**	0	0	10**
	Carcinoma, squamous cell		0	0	3*	0	0	1
Duodenum								
	Adenoma		0	0	1	0	0	0
Jejunum								
	Adenoma		0	0	1	0	0	2
	Adenocarcinoma		0	0	1	0	0	2
Ileum								
	Adenoma		0	0	1	0	0	1
	Hemangiosarcoma		0	0	1	0	0	0
Clitoral gland				[1]^a^				
	Carcinoma			1				
Ovary								
	Luteoma		1	0	0	0	0	0
	Hemangioma		0	0	0	0	2	1
Uterus								
	Endometrial adenoma		0	0	0	0	0	1
	Hemangioma		1	0	0	0	1	1
	Hemangiosarcoma		1	0	0	0	0	0
	Polyp, endometrial stromal		0	0	0	0	1	0
	Sarcoma, endometrial stromal		0	0	0	0	1	0
Musculature								
	Hemangioma		0	0	0	1	0	0
Skin/subcutis								
	Papilloma, squamous cell		1	0	1	1	0	1
	Hemangiosarcoma		0	1	0	0	1	0
Harderian gland								
	Adenoma		1	1	0	0	1	0
Brain								
	Hemangioma		0	0	0	0	1	0
Urethra					[2]^a^			[2]^a^
	Papilloma, transitional cell				2			2
All sites				[2]^a^	[10]^a^			[8]^a^
	Malignant lymphoma			2	10			8

Sham, sham‐exposed; MF Exp, magnetic field‐exposed; MNU, *N*‐methyl‐*N*‐nitrosourea‐treated.

^a^ Numbers in square brackets are for animals examined microscopically.

^*,**^Significant difference compared to the sham‐exposed group (*P *< 0.05, *P* < 0.01, respectively).

#### Non‐Neoplastic Lesions

The non‐neoplastic lesions observed in male and female mice are shown in supplementary Tables S5 and S6, respectively. In the MF‐ and sham‐exposed groups, high incidences of hyperplasia were observed in the adrenal subcapsular cells and in cystic endometrial cells of the uterus. Incidences of hyperplasia were observed at an intermediate frequency in the Harderian gland, thymic lymphocyte, and respiratory epithelium in the nasal cavity. Beside hyperplasia, frequent lesions included muscular myopathy, thymic involution, eosinophilic cytoplasmic change in the nasal cavity, eye cataract, and cellular infiltration of lymphocytes in the lacrimal gland. The frequency of these lesions was not significantly increased in the MF‐exposed groups compared to that in the sham‐exposed groups. In MNU‐treated groups, characteristic high incidences of atrophy of the retina, splenic and hepatic extramedullary hematopoiesis, and hyperplasia in the gastric squamous cells were observed, all of which were significantly more frequent than in the sham‐exposed groups.

## DISCUSSION

We evaluated the carcinogenicity of a 20 kHz IF‐MF in an approved short‐term bioassay using the Tg.rasH2 mouse model [Morton et al., 2002]. The experiment was performed in duplicate, with blinded histopathological examinations performed by a GLP‐licensed laboratory. The sham‐exposed mice bore spontaneous neoplastic as well as age‐related non‐neoplastic lesions with frequencies similar to those reported in the control data [Takaoka et al., 2003; Nambiar et al., 2012; Paranjpe et al., 2013a,2013b]. The MNU‐treated mice showed increased incidences of malignant lymphoma, gastric papillomas, and carcinomas, which were the typical incidences caused by MNU [Machida et al., 2008; Urano et al., 2012]. These baseline data indicate the validity of the present study. In the MF‐exposed mice, the observed neoplastic and non‐neoplastic lesions, gross pathological findings, and other measured parameters were mostly equivalent to those in the sham‐exposed mice, in their variety and frequency. No statistically significant, higher prevalence of neoplastic lesions was seen in the MF‐exposed groups compared to the sham‐exposed groups, demonstrating the absence of carcinogenicity in this mouse model.

One rare finding in the MF‐exposed group was a carcinoma of the clitoral gland. It is reported that the administration of acrylamide and 1,2‐dimethylhydrazine resulted in the formation of carcinoma in the clitoral gland in rats and mice, respectively [Turusov et al., 1994; Rice, 2005; NTP, 2012]. However, the results of our previous teratological [Nishimura et al., 2011], reproductive and developmental toxicity [Nishimura et al., 2012], acute/sub‐chronic toxicity [Nishimura et al., 2016], and other studies on IF‐MF exposure did not indicate the clitoral gland as a target organ of MF. Because this was an isolated incidence and was not reproduced in the present study, it is reasonable to assume that it occurred by chance and was unrelated to MF exposure.

In previous carcinogenicity studies of IF‐MF, Svedenstål and Holmberg [1993] exposed female CBA/S mice to a 20 kHz pulsed MF at 15 μT (peak‐to‐peak: pp) in sawtooth form, in combination with 5.24 Gy X‐ray irradiation. The survival rate of the MF‐exposed animals was significantly lower when compared to that of the control animals; however, the number of animals with lymphoma did not show any statistically significant difference between the control and MF‐alone‐exposed groups and between the X‐ray‐alone‐ and X‐ray plus MF‐exposed groups. Recently, Lee et al. [2007] conducted a series of chemically induced promotion studies on mammary, lung, and skin tumors in SD female rats, ICR male and female newborn mice, and young ICR female mice, respectively. They exposed the animals to a 20 kHz triangular MF (8 h/day) at 6.25 μT(rms) for 14, 6, or 20 weeks and found no promotional effect on tumors in either animal models.

Two genotoxicity studies [Miyakoshi et al., 2007; Sakurai et al., 2009] of 23 kHz MF exposure over 6 mT at the maximum were conducted using the Ames test, micronucleus formation, the comet assay, and hypoxanthine guanine phosphoribosyltransferase gene mutation assay; all assays gave negative results. The latest genotoxicity studies of a 90 kHz MF at 93.36 μT [Shi et al., 2014] and of a 7.5 kHz MF, 300 μT at the maximum [Herrala et al., 2018] by using the alkaline comet assay, the phosphorylated histone H2AX foci formation test, and others also resulted in negative outcomes. These previous studies together provided evidence for the lack of tumorigenic activity. The present study is in accordance with this negative evidence.

As described in the Materials and Methods section, the electric fields in various organs in our rasH2 mouse model, induced by the 0.20 mT MF at 20 kHz, were roughly four times more intense than the electric fields reported by Kumari et al. [2017], who exposed C57BL/6J mice to a 0.12 mT MF at 7.5 kHz. The maximum electric field induced in the present study (1.0 V/m in the skin) was approximately one‐third of the “basic restriction” for the general public (2.7 V/m at 20 kHz) of the ICNIRP guidelines [ICNIRP, 2010]. In our chick embryo studies [Nishimura et al., 2009, 2013], exposure to a 1.1 mT MF at 20 kHz induced a maximum of 1.80 V/m in the chick egg, which was calculated using Reilly's equation [Reilly, 1998]. These model‐based calculations are necessary to evaluate the compatibility of the MFs used for exposure experiments to the ICNIRP guidelines. However, it has not been proven that the induced electric field plays a pivotal role in toxicities or hazards that could be considered as significant health risks. Unless it is confirmed that health hazards are evoked through mechanisms in which the magnetically induced electric fields are involved, it is reasonable to consider that the reference flux density of MFs should be the primary dosimetry of exposure in health risk assessment.

## CONCLUSION

The results of the present study, conducted with duplicate experiments and involving blinded histopathological examinations, demonstrate a lack of carcinogenicity upon exposure of the rasH2 mouse model to a 20 kHz MF. The evidence presented in this study, along with that obtained in previous animal studies and associated genotoxicity studies regarding the effects of IF‐MFs, do not support the hypothesis that exposure to IF‐MFs is significantly carcinogenic.

## Supporting information

Additional supporting information may be found online in the Supporting Information section at the end of the article.

Supporting Table S1.

Supporting Table S2.

Supporting Table S3.

Supporting Table S4.

Supporting Table S5.

Supporting Table S6.
