# Supplementary material for: Carcinogenicity of intermediate frequency magnetic field in Tg.rasH2 mice
Source: Bioelectromagnetics. 2019 Mar 15;40(3):160–9. doi: 10.1002/bem.22177 (PMC6594107; doi:10.1002/bem.22177)
Supplement: Supplementary file 2 — Supporting Table S2. [file BEM-40-160-s002.doc]

TABLE S2. Gross pathology of female rasH2 mice exposed to a 20 kHz magnetic field

| Organ | Findings | Experiment | Experiment I | | | | | |  | Experiment II | | | | | |  |
| --- | --- | --- | --- | --- | --- | --- | --- | --- | --- | --- | --- | --- | --- | --- | --- | --- |
|  |  | Group | Sham | | MF Exp | | MNU | |  | Sham | | MF Exp | | MNU | |  |
|  |  | Dose | 0 mT | | 0.20 mT | | 75 mg/kg | |  | 0 mT | | 0.20 mT | | 75 mg/kg | |  |
| No. of animals/Group | | | 25 | [1]^a^ | 25 |  | 10 | [10]^a^ |  | 25 | [1]^a^ | 25 | [3]^a^ | 10 | [10]^a^ |  |
|  | | |  |  |  |  |  |  |  |  |  |  |  |  |  |  |
|  | Not remarkable | | 19 |  | 15 |  | 0 |  |  | 15 |  | 17 |  | 0 |  |  |
| Lymph node | | |  |  |  |  |  |  |  |  |  |  |  |  |  |  |
|  | Enlargement/Mandibular | | 0 |  | 0 |  | 1 | [1]^a^ |  | 0 |  | 0 |  | 5** | [5]^a^ |  |
|  | Enlargement/Pancreatic | | 0 |  | 0 |  | 1 | [1]^a^ |  | 0 |  | 0 |  | 5** | [5]^a^ |  |
|  | Enlargement/Mesenteric | | 0 |  | 1 |  | 2 | [2]^a^ |  | 0 |  | 0 |  | 4** | [4]^a^ |  |
|  | Enlargement/Renal | | 0 |  | 0 |  | 1 | [1]^a^ |  | 0 |  | 0 |  | 5** | [5]^a^ |  |
| Spleen | | |  |  |  |  |  |  |  |  |  |  |  |  |  |  |
|  | Enlargement | | 1 | [1]^a^ | 1 |  | 3 | [3]^a^ |  | 0 |  | 2 | [2]^a^ | 7** | [7]^a^ |  |
|  | Discolored area | | 2 |  | 4 |  | 1 | [1]^a^ |  | 4 |  | 1 |  | 2 | [2]^a^ |  |
| Thymus | | |  |  |  |  |  |  |  |  |  |  |  |  |  |  |
|  | Enlargement | | 0 |  | 2 |  | 6** | [6]^a^ |  | 0 |  | 0 |  | 8** | [8]^a^ |  |
|  | Adhered to heart | | 0 |  | 0 |  | 1 | [1]^a^ |  | 0 |  | 0 |  | 4** | [4]^a^ |  |
|  | Adhered to sternum | | 0 |  | 0 |  | 4** | [4]^a^ |  | 0 |  | 0 |  | 8** | [8]^a^ |  |
| Lung | | |  |  |  |  |  |  |  |  |  |  |  |  |  |  |
|  | Discolored spot | | 0 |  | 1 |  | 0 |  |  | 4 |  | 0* |  | 1 | [1]^a^ |  |
| Stomach | | |  |  |  |  |  |  |  |  |  |  |  |  |  |  |
|  | Nodule/Forestomach | | 0 |  | 2 |  | 10** | [10]^a^ |  | 0 |  | 0 |  | 10** | [10]^a^ |  |
| Small intestine | | |  |  |  |  |  |  |  |  |  |  |  |  |  |  |
|  | Intussusception | | 0 |  | 0 |  | 0 |  |  | 0 |  | 0 |  | 2 | [2]^a^ |  |
|  | Nodule | | 0 |  | 0 |  | 4** | [4]^a^ |  | 0 |  | 0 |  | 1 | [1]^a^ |  |
| Thoracic cavity | | |  |  |  |  |  |  |  |  |  |  |  |  |  |  |
|  | Contained fluid | | 0 |  | 0 |  | 5** | [5]^a^ |  | 1 | [1]^a^ | 0 |  | 4* | [4]^a^ |  |

Events less than 2/group throughout the experiments were omitted

Sham, sham-exposed; MF Exp, magnetic field-exposed; MNU, *N*-methyl-*N*-nitrosourea-treated

^a^: [*n*], number of dead animals

*, **: Significant difference compared to the sham-exposed group (*P* < 0.05, *P* < 0.01, respectively)
