# Supplementary material for: Carcinogenicity of intermediate frequency magnetic field in Tg.rasH2 mice
Source: Bioelectromagnetics. 2019 Mar 15;40(3):160–9. doi: 10.1002/bem.22177 (PMC6594107; doi:10.1002/bem.22177)
Supplement: Supplementary file 3 — Supporting Table S3. [file BEM-40-160-s003.doc]

TABLE S3. Organ weight data of rasH2 mice exposed to a 20 kHz magnetic field.

Experiment I

| Sex | Group | Dose | No. of animals examined | Body weight^a^ | Liver | Kidneys | Brain | Heart | Spleen | Lungs | Thymus | Testes  /Ovaries |
| --- | --- | --- | --- | --- | --- | --- | --- | --- | --- | --- | --- | --- |
|
| Male | Sham | 0 mT | 24 | 28.52 ± 2.83 | 1.142 ± 0.101 | 0.492 ± 0.049 | 0.467 ± 0.010 | 0.155 ± 0.013 | 0.058 ± 0.008 | 0.146 ± 0.009 | 0.036 ± 0.014 | 0.262 ± 0.036 |
|  | MF Exp | 0.20 mT | 25 | 29.20 ± 3.07 | 1.214 ± 0.085** | 0.531 ± 0.039** | 0.468 ± 0.015 | 0.166 ± 0.016** | 0.058 ± 0.009 | 0.154 ± 0.012** | 0.036 ± 0.014^b^ | 0.264 ± 0.022 |
|  |  |  |  |  |  |  |  |  |  |  |  |  |
| Female | Sham | 0 mT | 24 | 21.92 ± 1.28 | 1.021 ± 0.055 | 0.374 ± 0.025 | 0.496 ± 0.011 | 0.130 ± 0.010 | 0.075 ± 0.011 | 0.146 ± 0.017 | 0.039 ± 0.012 | 0.017 ± 0.002 |
|  | MF Exp | 0.20 mT | 25 | 21.23 ± 1.53* | 0.998 ± 0.111 | 0.380 ± 0.031 | 0.492 ± 0.017 | 0.131 ± 0.017 | 0.081 ± 0.043 | 0.146 ± 0.010 | 0.036 ± 0.017 | 0.018 ± 0.003 |

Experiment II

| Sex | Group | Dose | No. of animals examined | Body weight^a^ | Liver | Kidneys | Brain | Heart | Spleen | Lungs | Thymus | Testes  /Ovaries |
| --- | --- | --- | --- | --- | --- | --- | --- | --- | --- | --- | --- | --- |
|
| Male | Sham | 0 mT | 24 | 30.57 ± 2.96 | 1.204 ± 0.100 | 0.532 ± 0.051 | 0.477 ± 0.015 | 0.163 ± 0.016 | 0.060 ± 0.012 | 0.151 ± 0.011 | 0.039 ± 0.013 | 0.281 ± 0.023 |
|  | MF Exp | 0.20 mT | 23 | 29.69 ± 2.17 | 1.218 ± 0.068 | 0.531 ± 0.039 | 0.474 ± 0.014 | 0.164 ± 0.017 | 0.060 ± 0.011 | 0.151 ± 0.007 | 0.037 ± 0.009 | 0.273 ± 0.021 |
|  |  |  |  |  |  |  |  |  |  |  |  |  |
| Female | Sham | 0 mT | 24 | 22.61 ± 1.83 | 1.056 ± 0.089 | 0.375 ± 0.027 | 0.497 ± 0.011 | 0.126 ± 0.009 | 0.076 ± 0.028 | 0.140 ± 0.007 | 0.045 ± 0.014 | 0.018 ± 0.002 |
|  | MF Exp | 0.20 mT | 22 | 22.97 ± 1.31 | 1.078 ± 0.072 | 0.384 ± 0.021 | 0.498 ± 0.013 | 0.133 ± 0.009** | 0.075 ± 0.013 | 0.149 ± 0.041 | 0.044 ± 0.016 | 0.019 ± 0.002 |

Data presented as mean ± SD (g)

Sham, sham-exposed; MF Exp, magnetic field-exposed

^a^: Values obtained after overnight animal fasting

^b^: One organ was not measured due to human error

*, **: Significant difference compared to the sham-exposed group (*P* < 0.05, *P* < 0.01, respectively)
