# Supplementary material for: Carcinogenicity of intermediate frequency magnetic field in Tg.rasH2 mice
Source: Bioelectromagnetics. 2019 Mar 15;40(3):160–9. doi: 10.1002/bem.22177 (PMC6594107; doi:10.1002/bem.22177)
Supplement: Supplementary file 4 — Supporting Table S4. [file BEM-40-160-s004.doc]

TABLE S4. Organ to body weight ratio data of rasH2 mice exposed to a 20 kHz magnetic field

Experiment I

| Sex | Group | Dose | No. of animals examined |  | Liver | Kidneys | Brain | Heart | Spleen | Lungs | Thymus | Testes  /Ovaries |
| --- | --- | --- | --- | --- | --- | --- | --- | --- | --- | --- | --- | --- |
|
| Male | Sham | 0 mT | 24 |  | 4.012 ± 0.160 | 1.729 ± 0.123 | 1.652 ± 0.167 | 0.546 ± 0.043 | 0.205 ± 0.030 | 0.515 ± 0.034 | 0.127 ± 0.053 | 0.926 ± 0.139 |
|  | MF Exp | 0.20 mT | 25 |  | 4.177 ± 0.258** | 1.829 ± 0.138** | 1.617 ± 0.150 | 0.569 ± 0.048* | 0.199 ± 0.024 | 0.529 ± 0.042 | 0.120 ± 0.037^a^ | 0.910 ± 0.092 |
|  |  |  |  |  |  |  |  |  |  |  |  |  |
| Female | Sham | 0 mT | 24 |  | 4.665 ± 0.221 | 1.709 ± 0.101 | 2.267 ± 0.118 | 0.593 ± 0.038 | 0.342 ± 0.045 | 0.666 ± 0.054 | 0.178 ± 0.052 | 0.078 ± 0.009 |
|  | MF Exp | 0.20 mT | 25 |  | 4.693 ± 0.307 | 1.791 ± 0.077** | 2.326 ± 0.131 | 0.620 ± 0.099 | 0.389 ± 0.245 | 0.690 ± 0.050 | 0.166 ± 0.070 | 0.082 ± 0.012 |

Experiment II

| Sex | Group | Dose | No. of animals examined |  | Liver | Kidneys | Brain | Heart | Spleen | Lungs | Thymus | Testes  /Ovaries |
| --- | --- | --- | --- | --- | --- | --- | --- | --- | --- | --- | --- | --- |
|
| Male | Sham | 0 mT | 24 |  | 3.949 ± 0.223 | 1.747 ± 0.143 | 1.574 ± 0.149 | 0.535 ± 0.051 | 0.194 ± 0.033 | 0.496 ± 0.032 | 0.128 ± 0.040 | 0.926 ± 0.098 |
|  | MF Exp | 0.20 mT | 23 |  | 4.111 ± 0.166** | 1.790 ± 0.109 | 1.604 ± 0.103 | 0.554 ± 0.049 | 0.203 ± 0.035 | 0.511 ± 0.033 | 0.125 ± 0.030 | 0.925 ± 0.104 |
|  |  |  |  |  |  |  |  |  |  |  |  |  |
| Female | Sham | 0 mT | 24 |  | 4.676 ± 0.201 | 1.660 ± 0.066 | 2.208 ± 0.145 | 0.559 ± 0.038 | 0.334 ± 0.106 | 0.622 ± 0.038 | 0.197 ± 0.063 | 0.081 ± 0.011 |
|  | MF Exp | 0.20 mT | 22 |  | 4.695 ± 0.215 | 1.676 ± 0.107 | 2.171 ± 0.110 | 0.579 ± 0.041* | 0.326 ± 0.047 | 0.646 ± 0.150 | 0.190 ± 0.064 | 0.085 ± 0.011 |

Data presented as mean ± SD (g/100 g body weight)

Sham, sham-exposed; MF Exp, magnetic field-exposed

^a^: One organ was not measured due to human error

*, **: Significant difference compared to the sham-exposed group (*P* < 0.05, *P* < 0.01, respectively)
