# Supplementary material for: Carcinogenicity of intermediate frequency magnetic field in Tg.rasH2 mice
Source: Bioelectromagnetics. 2019 Mar 15;40(3):160–9. doi: 10.1002/bem.22177 (PMC6594107; doi:10.1002/bem.22177)
Supplement: Supplementary file 5 — Supporting Table S5. [file BEM-40-160-s005.doc]

TABLE S5. Non-neoplastic lesions of male rasH2 mice exposed to a 20 kHz magnetic field

| Organ | Findings | Experiment | Experiment I | | | | | |  | Experiment II | | | | | |  |
| --- | --- | --- | --- | --- | --- | --- | --- | --- | --- | --- | --- | --- | --- | --- | --- | --- |
|  |  | Group | Sham | | MF Exp | | MNU | |  | Sham | | MF Exp | | MNU | |  |
|  |  | Dose | 0 mT | | 0.20 mT | | 75 mg/kg | |  | 0 mT | | 0.20 mT | | 75 mg/kg | |  |
| No. of animals/Group | | | 25 |  | 25 |  | 10 |  |  | 25 |  | 25 |  | 10 |  |  |
| Mandibular lymph node | | |  |  |  |  |  |  |  |  |  |  |  |  |  |  |
|  | Cellular infiltration, histiocyte | | 2 |  | 4 |  | 0 |  |  | 1 |  | 0 |  | 0 |  |  |
| Mesenteric lymph node | | |  |  |  |  |  |  |  |  |  |  |  |  |  |  |
|  | Dilatation, sinus | | 2 |  | 3 |  | 3 |  |  | 0 |  | 0 |  | 0 |  |  |
| Spleen | | |  |  |  |  |  |  |  |  |  |  |  |  |  |  |
|  | Extramedullary hematopoiesis | | 0 |  | 0 |  | 6** |  |  | 0 |  | 0 |  | 3** |  |  |
|  | Pigmentation | | 2 |  | 5 |  | 1 |  |  | 1 |  | 3 |  | 1 |  |  |
| Bone marrow | | |  |  |  |  |  |  |  |  |  |  |  |  |  |  |
|  | Hyperplasia, myeloid | | 0 |  | 0 |  | 1 |  |  | 0 |  | 0 |  | 2 |  |  |
| Thymus | | |  |  |  |  |  |  |  |  |  |  |  |  |  |  |
|  | Apoptosis | | 0 |  | 0 |  | 0 |  |  | 0 |  | 0 |  | 2* |  |  |
|  | Atrophy | | 0 |  | 0 |  | 1 |  |  | 0 |  | 0 |  | 2* |  |  |
|  | Involution | | 7 |  | 6 |  | 1 |  |  | 6 |  | 7 |  | 0 |  |  |
|  | Hyperplasia, lymphocyte | | 2 |  | 5 |  | 0 |  |  | 2 |  | 2 |  | 0 |  |  |
| Parathyroid | | |  |  |  |  |  |  |  |  |  |  |  |  |  |  |
|  | Cyst | | 3 |  | 1 |  | 3 |  |  | 1 |  | 2 |  | 1 |  |  |
| Adrenal | | |  |  |  |  |  |  |  |  |  |  |  |  |  |  |
|  | Hyperplasia, subcapsular cell | | 9 |  | 8 |  | 4 |  |  | 4 |  | 3 |  | 2 |  |  |
| Nasal cavity | | |  |  |  |  |  |  |  |  |  |  |  |  |  |  |
|  | Eosinophilic cytoplasmic change | | 7 |  | 6 |  | 2 |  |  | 4 |  | 10 |  | 4 |  |  |
|  | Hyperplasia, respiratory epithelium | | 1 |  | 3 |  | 3 |  |  | 1 |  | 3 |  | 1 |  |  |
| Lung/bronchial | | |  |  |  |  |  |  |  |  |  |  |  |  |  |  |
|  | Hyperplasia, bronchiolo-alveolar | | 1 |  | 0 |  | 2 |  |  | 0 |  | 0 |  | 1 |  |  |
| Tongue | | |  |  |  |  |  |  |  |  |  |  |  |  |  |  |
|  | Hyperplasia, squamous cell | | 0 |  | 0 |  | 2 |  |  | 0 |  | 0 |  | 1 |  |  |
| Salivary gland | | |  |  |  |  |  |  |  |  |  |  |  |  |  |  |
|  | Atrophy, acinar cell | | 0 |  | 0 |  | 0 |  |  | 0 |  | 0 |  | 2* |  |  |
|  | Cellular infiltration, lymphocyte | | 2 |  | 1 |  | 0 |  |  | 0 |  | 1 |  | 0 |  |  |
| Stomach | | |  |  |  |  |  |  |  |  |  |  |  |  |  |  |
|  | Hyperplasia, glandular cell | | 1 |  | 2 |  | 2 |  |  | 0 |  | 0 |  | 0 |  |  |
|  | Hyperplasia, squamous cell | | 0 |  | 1 |  | 3* |  |  | 0 |  | 0 |  | 6** |  |  |
| Jejunum | | |  |  |  |  |  |  |  |  |  |  |  |  |  |  |
|  | Hyperplasia | | 0 |  | 0 |  | 2 |  |  | 0 |  | 0 |  | 0 |  |  |
| Liver | | |  |  |  |  |  |  |  |  |  |  |  |  |  |  |
|  | Microgranuloma | | 4 |  | 2 |  | 1 |  |  | 1 |  | 2 |  | 0 |  |  |
| Kidney | | |  |  |  |  |  |  |  |  |  |  |  |  |  |  |
|  | Cellular infiltration, lymphocyte | | 2 |  | 0 |  | 0 |  |  | 0 |  | 0 |  | 0 |  |  |
| Musculature | | |  |  |  |  |  |  |  |  |  |  |  |  |  |  |
|  | Myopathy | | 20 |  | 16 |  | 7 |  |  | 22 |  | 16* |  | 5* |  |  |
| Eye | | |  |  |  |  |  |  |  |  |  |  |  |  |  |  |
|  | Atrophy of retina | | 0 |  | 0 |  | 8** |  |  | 0 |  | 0 |  | 8** |  |  |
|  | Cataract | | 8 |  | 8 |  | 4 |  |  | 4 |  | 3 |  | 0 |  |  |
| Harderian gland | | |  |  |  |  |  |  |  |  |  |  |  |  |  |  |
|  | Cellular infiltration, lymphocyte | | 3 |  | 0 |  | 0 |  |  | 0 |  | 0 |  | 0 |  |  |
|  | Hyperplasia | | 5 |  | 5 |  | 0 |  |  | 0 |  | 0 |  | 1 |  |  |
| Lacrimal gland | | |  |  |  |  |  |  |  |  |  |  |  |  |  |  |
|  | Cellular infiltration, lymphocyte | | 5 |  | 7 |  | 1 |  |  | 6 |  | 6 |  | 0 |  |  |
|  | Inflammation | | 3 |  | 0 |  | 0 |  |  | 0 |  | 0 |  | 0 |  |  |

Events less than 2/group throughout the experiments were omitted

Sham, sham-exposed

MF Exp, magnetic field-exposed

MNU, *N*-methyl-*N*-nitrosourea-treated

*, **: Significant difference compared to the sham-exposed group (*P* < 0.05, *P* < 0.01, respectively)
